# A Clinical Prediction Model to Predict Heparin Treatment Outcomes and Provide Dosage Recommendations: Development and Validation Study

**DOI:** 10.2196/27118

**Published:** 2021-05-20

**Authors:** Dongkai Li, Jianwei Gao, Na Hong, Hao Wang, Longxiang Su, Chun Liu, Jie He, Huizhen Jiang, Qiang Wang, Yun Long, Weiguo Zhu

**Affiliations:** 1 Department of Critical Care Medicine, State Key Laboratory of Complex Severe and Rare Diseases, Peking Union Medical College Hospital, Peking Union Medical College, Chinese Academy of Medical Sciences Beijing China; 2 Digital Health China Technologies Co. Ltd. Beijing China; 3 Department of Information Center, State Key Laboratory of Complex Severe and Rare Diseases, Peking Union Medical College Hospital, Chinese Academy of Medical Sciences and Peking Union Medical College Beijing China; 4 Department of Primary Care and Family Medicine, State Key Laboratory of Complex Severe and Rare Diseases, Peking Union Medical College Hospital, Chinese Academy of Medical Sciences and Peking Union Medical College Beijing China

**Keywords:** outcome prediction, clinical decision support, dosage recommendation, machine learning, intensive care unit

## Abstract

**Background:**

Unfractionated heparin is widely used in the intensive care unit as an anticoagulant. However, weight-based heparin dosing has been shown to be suboptimal and may place patients at unnecessary risk during their intensive care unit stay.

**Objective:**

In this study, we intended to develop and validate a machine learning–based model to predict heparin treatment outcomes and to provide dosage recommendations to clinicians.

**Methods:**

A shallow neural network model was adopted in a retrospective cohort of patients from the Multiparameter Intelligent Monitoring in Intensive Care III (MIMIC III) database and patients admitted to the Peking Union Medical College Hospital (PUMCH). We modeled the subtherapeutic, normal, and supratherapeutic activated partial thromboplastin time (aPTT) as the outcomes of heparin treatment and used a group of clinical features for modeling. Our model classifies patients into 3 different therapeutic states. We tested the prediction ability of our model and evaluated its performance by using accuracy, the kappa coefficient, precision, recall, and the F1 score. Furthermore, a dosage recommendation module was designed and evaluated for clinical decision support.

**Results:**

A total of 3607 patients selected from MIMIC III and 1549 patients admitted to the PUMCH who met our criteria were included in this study. The shallow neural network model showed results of F1 scores 0.887 (MIMIC III) and 0.925 (PUMCH). When compared with the actual dosage prescribed, our model recommended increasing the dosage for 72.2% (MIMIC III, 1240/1718) and 64.7% (PUMCH, 281/434) of the subtherapeutic patients and decreasing the dosage for 80.9% (MIMIC III, 504/623) and 76.7% (PUMCH, 277/361) of the supratherapeutic patients, suggesting that the recommendations can contribute to clinical improvements and that they may effectively reduce the time to optimal dosage in the clinical setting.

**Conclusions:**

The evaluation of our model for predicting heparin treatment outcomes demonstrated that the developed model is potentially applicable for reducing the misdosage of heparin and for providing appropriate decision recommendations to clinicians.

## Introduction

Existing rule-based protocols guide clinicians to initiate, modulate, or terminate a certain treatment procedure according to evidence-based clinical guidelines or best practices [1]. However, in real clinical scenarios, dynamic changes occur continuously in individual patients with complex diseases and physiological situations, which frequently exceed the scope of the typical model described in the guidelines [2]. In particular, in critical care settings, there is a decision-making dilemma between evidence-based medicine and individualized medicine due to the lack of high-quality evidence and the urgency for the accuracy and effectiveness of the treatment [3]. Fortunately, in the era of big data and artificial intelligence and based on progress in data acquisition, integration, and application in critical care medicine, machine learning techniques sometimes can help with diagnosis, treatment, and prediction in intensive care units (ICUs) [4-6].

Heparin (or unfractionated heparin, UFH) infusion is one example of a medication for which retrospective data have been proven to provide valuable support in clinical settings, especially in critical settings in which patients are vulnerable to thromboembolism or hemorrhage or to severe complications caused by these conditions [7]. For decades, UFH dosing has been based solely on a patient’s weight: a weight-based heparin dosing nomogram is the standard practice for the application of UFH. The heparin nomogram mainly consists of an empirical initial loading dose followed by a step-by-step modulation according to a series of blood clotting parameters that are monitored every 4-6 hours [8,9]. The actual optimal UFH dosage varies widely among patients with different physiological situations, and for these patients, the time to optimal dosage may be prolonged. Besides, adverse events associated with supratherapeutic or subtherapeutic anticoagulation, such as hemorrhagic tendency or thrombophilia, may occur due to the intravenous heparin’s narrow therapeutic window [7]. Therefore, retrospective analysis starts by extracting sequential dose response data as well as the concurrent laboratory and other clinical data, which may greatly contribute to the improvement on the feedback delay of UFH dosage optimization [10]. In our recent study [11], based on the public Multiparameter Intelligent Monitoring in Intensive Care III (MIMIC III) and electronic ICU databases [12,13], we compared several common models for predicting the effects of heparin treatment and showed that machine learning–based models, which outperformed the standard practices, can be used to optimize and personalize heparin dosing to improve patient safety.

Although our UFH model has been evaluated and validated using public databases [10,14], it has not been validated in our local clinical setting, especially for step-by-step modulation. In this study, we extend the validation and application of the previously optimized UFH model to a local clinical database in the Department of Critical Care Medicine of Peking Union Medical College Hospital (PUMCH), which is a complementary ICU in a tertiary hospital in China. Furthermore, UFH dosage recommendation based on the machine learning model was also performed for both the MIMIC and PUMCH databases, with the goal of effectively reducing the time to optimal dosage in the clinical setting. Finally, to further promote understanding of the data model and algorithm, we performed a featured importance analysis in both databases.

## Methods

### Data Sets

To evaluate the adaptability of the predictive model and to implement external validation, we employed patient data, including heparin treatments from 2 databases: the MIMIC III database and the PUMCH ICU database. The MIMIC III database is a free and open intensive care medical data set published by the Computational Physiology Laboratory of the Massachusetts Institute of Technology, the Beth Israel Deaconess Medical Center, and Philips Healthcare. It contains real medical data from more than 50,000 adult patients in the ICU at the Beth Israel Deaconess Medical Center between 2001 and 2012 [8]. The PUMCH ICU database comprises the complete clinical data of patients admitted to the PUMCH ICU with a retrospective cohort of more than 20,000 ICU patients between 2013 and 2019.

### Definition of Heparin Treatment Outcomes

**Figure 1:**
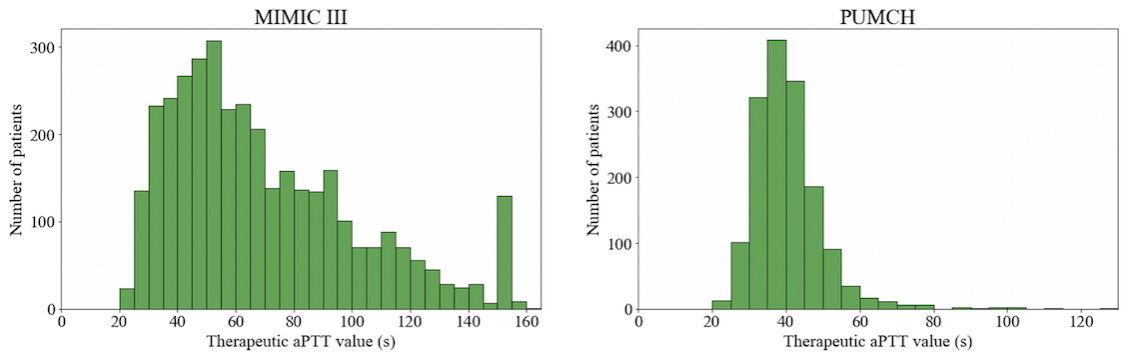
The therapeutic activated partial thromboplastin time distributions in the 2 data sets. aPTT: activated partial thromboplastin time; MIMIC III: Multiparameter Intelligent Monitoring in Intensive Care III; PUMCH: Peking Union Medical College Hospital.

We classified patients as subtherapeutic, normal therapeutic, and supratherapeutic according to their therapeutic activated partial thromboplastin time (aPTT) values after heparin treatment. We used the average aPTT value from 8 h to 24 h after the initial heparin infusion as the therapeutic aPTT value. Figure 1 shows the distributions of the aPTT values in the 2 data sets. Due to differences in the regions, patient characteristics, and treatment plans such as the step-by-step treatment pattern of PUMCH, the observed therapeutic aPTT distributions are quite different. Based on a previous study [14] and suggestions from clinicians, we adopted different definition ranges for the 2 data sets (Table 1). We then labelled each patient record with 1 of the 3 labels (subtherapeutic, normal therapeutic, and supratherapeutic), thereby converting the clinical outcome prediction task into a ternary classification task.

**Table 1:** Ranges of the therapeutic activated partial thromboplastin time classification of the 2 data sets.

Activated partial thromboplastin time (time in seconds)	Multiparameter Intelligent Monitoring in Intensive Care III data set	Peking Union Medical College Hospital data set
Subtherapeutic activated partial thromboplastin time (s)	0-60	0-35
Normal therapeutic activated partial thromboplastin time (s)	60-100	35-45
Supratherapeutic activated partial thromboplastin time (s)	>100	>45

### Feature Selection

According to evidence from related studies and experience from clinical practice, various clinical features affect heparin treatment outcomes [14], that is, the therapeutic aPTT. For example, creatinine in the blood is almost entirely filtered into the urine via glomerular filtration and its concentration is stable under normal circumstances. Therefore, creatinine concentration in the blood can be used as an indicator of renal function because it reflects the filtration function of glomeruli. Measurements of renal, hepatic, cardiac, and coagulation functions were included as features of interest. Aspartate aminotransferase (AST) and alanine aminotransferase (ALT) concentrations in the blood are sensitive to hepatocellular damage, and their ratio is an important indicator of liver function. Sequential organ failure assessment (SOFA) scores were included. Furthermore, the total heparin dosage, defined as the sum of the heparin doses administered within 8 h of the initial heparin infusion, is also considered as the affected factor of heparin treatment outcomes. Therefore, to optimize the model predictions, we selected clinical features of interest from the 2 data sets, including sex, ethnicity, admission type, age, weight, initial aPTT, creatinine, AST/ALT ratio (we used the ALT value instead for PUMCH since AST values were not routinely tested every time in PUMCH), several SOFA scores, and total heparin dosage, as shown in Table 2. We used the last aPTT measurement before heparin treatment as the initial aPTT value, and the laboratory tests and SOFA scores were those closest to the initial heparin injection time.

**Table 2:** Clinical features of interest chosen from the 2 data sets.

Features	Multiparameter Intelligent Monitoring in Intensive Care III data set	Peking Union Medical College Hospital data set
**Demographic data**		
	Gender	Gender
	Ethnicity	—^a^
	Admission type	—
	Age	Age
	Weight	Weight
**Laboratory tests**		
	Initial aPTT^b^ value	Initial aPTT value
	Creatinine value	Creatinine value
	AST/ALT^c^ ratio	Alanine aminotransferase value
**SOFA^d^ scores**		
	Coagulation SOFA score	Coagulation SOFA score
	Liver SOFA score	Liver SOFA score
	Cardiovascular SOFA score	Cardiovascular SOFA score
	Renal SOFA score	Renal SOFA score
Medication	Total heparin dosage	Total heparin dosage

^a^Not available.

^b^aPTT: activated partial thromboplastin time.

^c^AST/ALT: aspartate aminotransferase/alanine aminotransferase.

^d^SOFA: sequential organ failure assessment.

### Patient Inclusion Criteria

**Figure 2:**
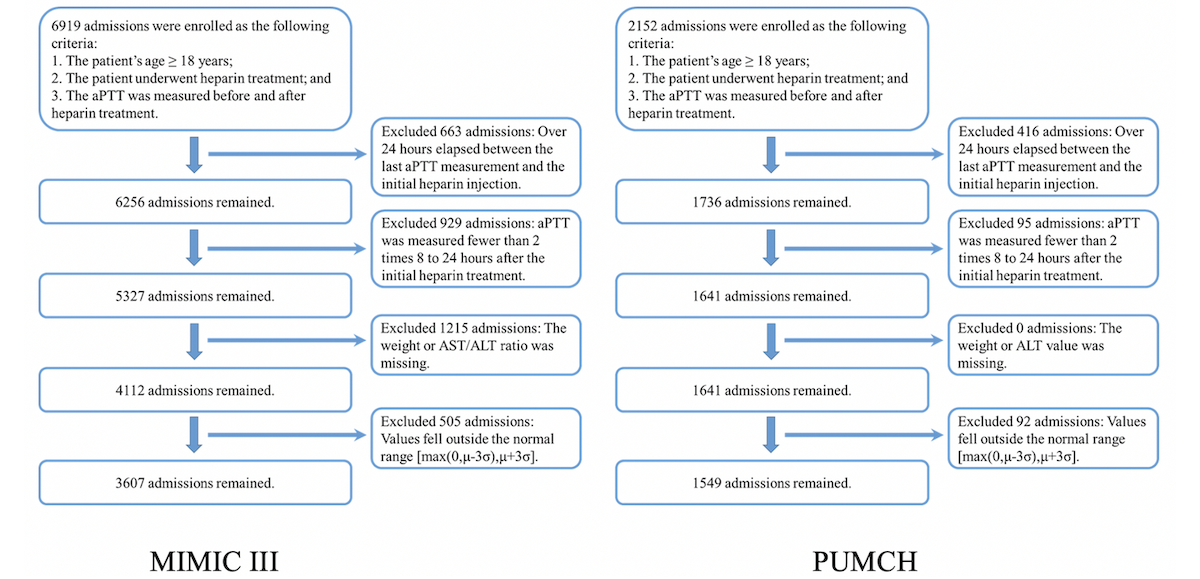
Study cohort selection workflow based on the inclusion and exclusion criteria. ALT: alanine aminotransferase; aPTT: activated partial thromboplastin time; AST: aspartate aminotransferase; MIMIC III: Multiparameter Intelligent Monitoring in Intensive Care III; PUMCH: Peking Union Medical College Hospital.

The enrollment criteria were as follows: (1) patient’s age ≥18 years, (2) patient underwent heparin treatment, and (3) the aPTT value was measured before and after heparin treatment. Based on the above criteria, we initially collected 6919 patient records from the MIMIC III database and 2152 patient records from the PUMCH database. For the MIMIC III database, we first removed some patient records whose aPTT data were unavailable. We then removed records with missing values for weight or the ratio of AST/ALT. Next, we removed patient records with values of continuous features outside the normal ranges, including weight, initial aPTT value, creatinine value, AST/ALT ratio, and total heparin dosage. According to the statistical definition of outliers [15], we calculated the mean value μ and standard deviation σ of these features; the normal range includes values from max(0, μ-3σ) to μ+3σ. We therefore removed the individual records outside of this range. The normal ranges and number of outliers for each feature are listed in Multimedia Appendix 1. After this process, 3607 patient records remained. For the PUMCH database, after a similar data selection process (according to different recording methods such as the replacement of the AST/ALT ratio by the ALT value in the PUMCH database), we collected 1549 patient records as the study population of PUMCH. The details of the selection process are shown in Figure 2.

### Data Preprocessing

SOFA scores were missing in both data sets, and the 3 nearest neighbors algorithms were used to substitute the missing value with the mean of the 3 neighbors that was closest in terms of the Euclidean distance. The missing data imputation results are listed in Multimedia Appendix 2. We applied a one-hot encoder for the gender, ethnicity, and admission type features since these are categorical features. To avoid the occurrence of feature values that are too large for model training, we applied min-max normalization to all the continuous numerical features using the following formula:


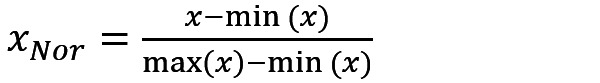


where x denotes the values of all records for a fixed feature.

### Model Training

As validated in a previous study [11], a shallow neural network model works best from among several machine learning models for the heparin outcome prediction task. In this study, we used a fully connected shallow neural network model [16,17] to predict the therapeutic effect (subtherapeutic, normal therapeutic, or supratherapeutic) of patients after 8 hours of heparin treatment. This is a ternary classification task. Our artificial neural network consists of an input layer, an output layer, and 3 hidden layers. It is fully connected, which means that each neuron receives input from all the neurons in the previous layer and passes on the results to all the neurons in the next layer. The rectified linear unit function [18] was used as the activation function in order to increase the nonlinearity and improve the model efficiency. The number of neurons in the hidden layers was set at 32/64/24. The models for MIMIC III and PUMCH data sets are basically the same, except for slight differences in the input. We trained the model for 5000 epochs with a learning rate of 0.015. The fully connected neural network was built using TensorFlow (version 1.12.0). Each data set was divided into training (80%) and test (20%) sets, with the proportion of subtherapeutic records, normal therapeutic records, and supratherapeutic records being maintained. To validate the predictive performance of our model, 5-fold cross-validation was used on each data set.

### Heparin Dosage Recommendation

By using the neural network model described above, we can calculate the probability that a record belongs to each of the 3 categories (subtherapeutic, normal therapeutic, and supratherapeutic) with the softmax function [19]. For a subtherapeutic or supratherapeutic patient record x and a possible dosage α, we change the actual total heparin dosage of x to α and defined M(x,α) to be the normal therapeutic probability after this changing. Then, we calculated M(x,α) by the softmax function shown below:


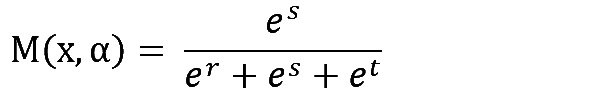


where r, s, and t are the outputs (a larger number means a higher probability of belonging to this category) of the neural network model for supratherapeutic, normal therapeutic, and supratherapeutic, respectively, after this changing.

For this subtherapeutic or supratherapeutic patient record x, we provided a recommended total heparin dosage σ(x) for x, which maximized the normal therapeutic probability. More specifically, we computed the recommended dosage using the following formula:


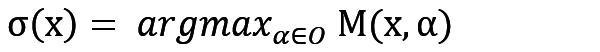


where O denotes the set of all possible dosages. We believe that our recommended dosage is reasonable and that our model may improve the clinician’s judgement.

### Model Evaluation

#### Model Performance Evaluation

The following measures, that is, precision, recall, F1 score, accuracy, and the kappa coefficient, were used to evaluate the capability of our ternary classification model [20,21]. Because the microaveraged precision, recall, and F1 score are all equal to accuracy, we only computed the accuracy, kappa coefficient, macroaveraged precision, recall, and F1 score to gauge the classification performance. For details, see the confusion matrix in Table 3 and the formulas below.

**Table 3:** The confusion matrix of our prediction.^a^

Actual label	Prediction label		
	Subtherapeutic	Normal therapeutic	Supratherapeutic
Subtherapeutic	A	B	C
Normal therapeutic	D	E	F
Supratherapeutic	G	H	I

^a^This table was used to gauge the classification performance, as shown in the formulas.


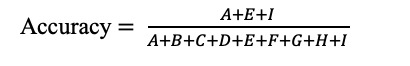



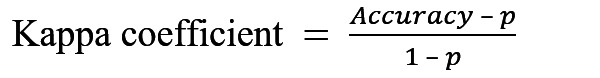



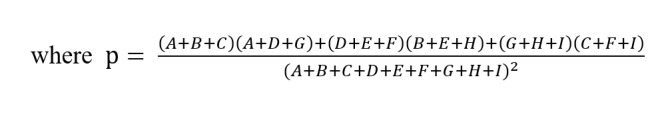



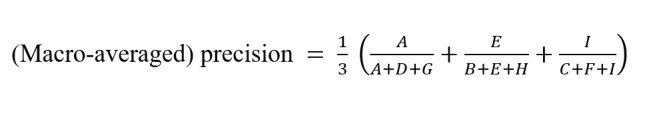



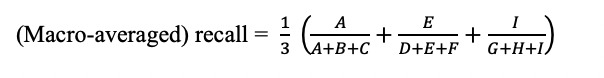



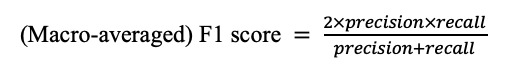


#### Feature Importance Evaluation

To evaluate the importance of each feature in our shallow neural network model, we removed 1 feature at a time and then calculated the decline in the model accuracy. More specifically, the importance *E_i_* of the i-th feature *F_i_* can be calculated by the formula *E_i_ = A – A_i_,* where is the model accuracy when all features are used and *A_i_* is the model accuracy after removing feature *F_i_*. The features with higher *E_i_* values are more important to the model. We also used 5-fold cross-validation on each data set to improve the stability of the calculation.

#### Recommendation Result Evaluation

The recommendation dosage result was compared with the actual decision made by the clinicians in the retrospective database, and the corresponding dosage response in the clinical scenario was also evaluated to validate whether the actual dosage setting was optimal. When the actual dosage setting was subtherapeutic and our recommended dosage was higher or it was supratherapeutic and our recommended dosage was lower, then we believe our recommended dosage was reasonable. For example, for a patient with predicted subtherapeutic aPTT outcomes after 10,000 IUs of heparin treatment, if a model recommended that dosage is greater than 10,000 IUs, we considered the recommended dosage to be reasonable; if the model recommended dosage is less than 10,000 IUs, we considered the recommended dosage to be unreasonable.

## Results

### Patient Characteristics

Patient characteristics according to the therapeutic outcome after heparin injection in MIMIC III and PUMCH are shown in Table 4. Among the 3607 MIMIC III patients, 1718 (47.6%) showed aPPT values within the subtherapeutic range, 1266 (35.1%) had values within the normal therapeutic range, and 623 (17.3%) patients had values within the supratherapeutic range. Among the 1549 PUMCH patients, 434 (28.1%) had measured aPPT values within the subtherapeutic range, 754 (48.7%) had values within the normal therapeutic range, and 361 (23.3%) had values within the supratherapeutic range. For numeric features, the feature density distribution was generated to clarify whether the values were scattered or centered (Multimedia Appendix 3).

**Table 4:** Patient characteristics and selected features according to the therapeutic outcome after heparin injection.

Data set, patient characteristics				Therapeutic range category				
				Subtherapeutic		Normal therapeutic		Supratherapeutic
**Multiparameter Intelligent Monitoring in Intensive Care III database (n=3607)**				1718		1266		623
	Age (years), mean (SD)			65.4 (14.2)		67.8 (15.0)		70.6 (13.9)
	Weight (kg), mean (SD)			84.5 (21.8)		81.3 (21.3)		80.5 (21.8)
	**Gender, n (%)**							
		Male	1068 (62.2)		750 (59.2)		320 (51.4)	
		Female	650 (37.8)		516 (40.8)		303 (48.6)	
	**Ethnicity, n (%)**							
		White	1223 (71.2)		928 (73.3)		441 (70.8)	
		Black	98 (5.7)		111 (8.8)		71 (11.4)	
		Latin	42 (2.4)		21 (1.7)		16 (2.6)	
		Asian	24 (1.4)		25 (2.0)		25 (4.0)	
		Others	331 (19.3)		181 (14.3)		70 (11.2)	
	**Admission type, n (%)**							
		Elective	189 (11.0)		69 (5.5)		22 (3.5)	
		Emergency	1474 (85.8)		1152 (91.0)		591 (94.9)	
		Urgent	55 (3.2)		45 (3.6)		10 (1.6)	
	Initial aPTT^a^ value (s), mean (SD)			39.5 (22.0)		45.5 (26.8)		40.4 (21.2)
	Creatinine value (mg/dL), mean (SD)			1.4 (1.0)		1.5 (1.1)		1.7 (1.2)
	AST/ALT^b^ ratio, mean (SD)			1.6 (1.1)		1.7 (1.2)		1.6 (1.0)
	Coagulation SOFA^c^ score, mean (SD)			0.5 (0.8)		0.4 (0.7)		0.4 (0.7)
	Liver SOFA score, mean (SD)			0.4 (0.8)		0.4 (0.7)		0.4 (0.8)
	Cardiovascular SOFA score, mean (SD)			1.5 (1.2)		1.5 (1.3)		1.7 (1.3)
	Renal SOFA score, mean (SD)			0.8 (1.1)		1.1 (1.2)		1.3 (1.2)
	Total heparin dosage (IUs), mean (SD)			8449.7 (6773.0)		11299.9 (7550.8)		12667.3 (6932.3)
**Peking Union Medical College Hospital (n=1549)**				434		754		361
	Age (years), mean (SD)			55.1 (15.9)		57.8 (15.5)		60.9 (14.9)
	Weight (kg), mean (SD)			68.1 (12.6)		67.3 (12.2)		66.5 (12.4)
	**Gender, n (%)**							
		Male	256 (59.0)		453 (60.1)		223 (61.8)	
		Female	178 (41.0)		301 (39.9)		138 (38.2)	
	Initial aPTT value (s), mean (SD)			29.5 (5.5)		34.6 (5.8)		39.4 (9.2)
	Creatinine value (μmol/L), mean (SD)			107.4 (73.7)		117.2 (78.7)		128.0 (86.3)
	Alanine aminotransferase value (unit/L), mean (SD)			45.1 (91.7)		47.1 (104.5)		55.7 (141.9)
	Coagulation SOFA score, mean (SD)			0.9 (0.9)		1.0 (1.0)		1.1 (1.0)
	Liver SOFA score, mean (SD)			0.6 (0.8)		0.7 (0.9)		0.8 (1.0)
	Cardiovascular SOFA score, mean (SD)			2.9 (1.6)		3.0 (1.5)		3.1 (1.5)
	Renal SOFA score, mean (SD)			0.5 (0.9)		0.6 (1.0)		0.8 (1.0)
	Total heparin dosage (IU/kg), mean (SD)			7.5 (4.3)		7.8 (4.4)		9.6 (5.6)

^a^aPTT: activated partial thromboplastin time.

^b^AST/ALT: aspartate aminotransferase/alanine aminotransferase.

^c^SOFA: sequential organ failure assessment.

### Model Performance

We divided each data set into a training (80%) set and test (20%) set. The proportions of each therapeutic level after dividing the data sets are listed in Multimedia Appendix 4. The shallow neural network model was trained and tested on a retrospective cohort of MIMIC III and PUMCH. The model performance results are listed in Table 5. For both data sets, our model achieved an accuracy of over 0.89, a kappa coefficient of over 0.82, and a macroaveraged F1 score of over 0.88. The results reflect the stable performance of the fully connected shallow neural network model and suggest that our model has generalizability for different databases.

**Table 5:** Model performance on the 2 data sets.

Parameters	Multiparameter Intelligent Monitoring in Intensive Care III data set	Peking Union Medical College Hospital data set
Accuracy	0.891	0.926
Kappa coefficient	0.823	0.882
Macroaveraged precision	0.890	0.931
Macroaveraged recall	0.884	0.920
Macroaveraged F1 score	0.887	0.925

### Feature Importance

**Figure 3:**
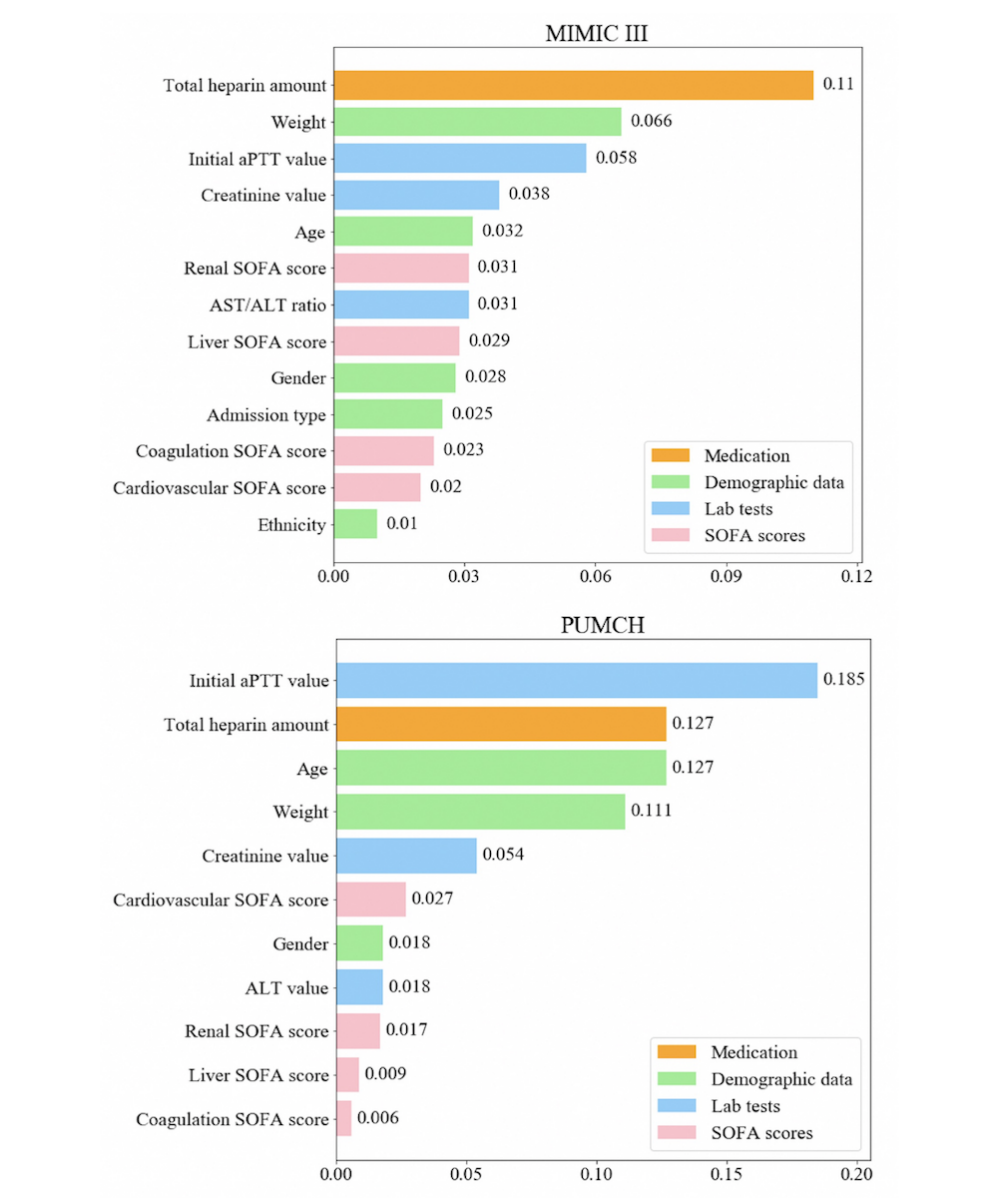
Feature importance. ALT: alanine aminotransferase; aPTT: activated partial thromboplastin time; AST: aspartate aminotransferase; MIMIC III: Multiparameter Intelligent Monitoring in Intensive Care III; PUMCH: Peking Union Medical College Hospital; SOFA: sequential organ failure assessment.

Figure 3 illustrates the importance of each feature in the development of our predictive model. The features are colored group-wise according to the categories listed in Table 2. A higher value indicates greater significance of the feature in the model. From the feature importance evaluation results, we conclude that the key features are basically the same in the 2 data sets but that their rankings differ slightly. For the MIMIC III data set, the total heparin dosage is the most influential model factor; weight, initial aPTT value, creatinine value, and age are key features and contributed substantially to the model. For the PUMCH data set, the highest impact feature is the initial aPTT value; total heparin dosage, age, weight, and creatinine are also important features for construction of the model. Ethnicity, SOFA scores, and gender have relatively small effects on the model in both data sets. Overall, the feature contribution analysis results are relatively consistent with clinical experience.

### Evaluation Results of Recommended Heparin Dosage

**Figure 4:**
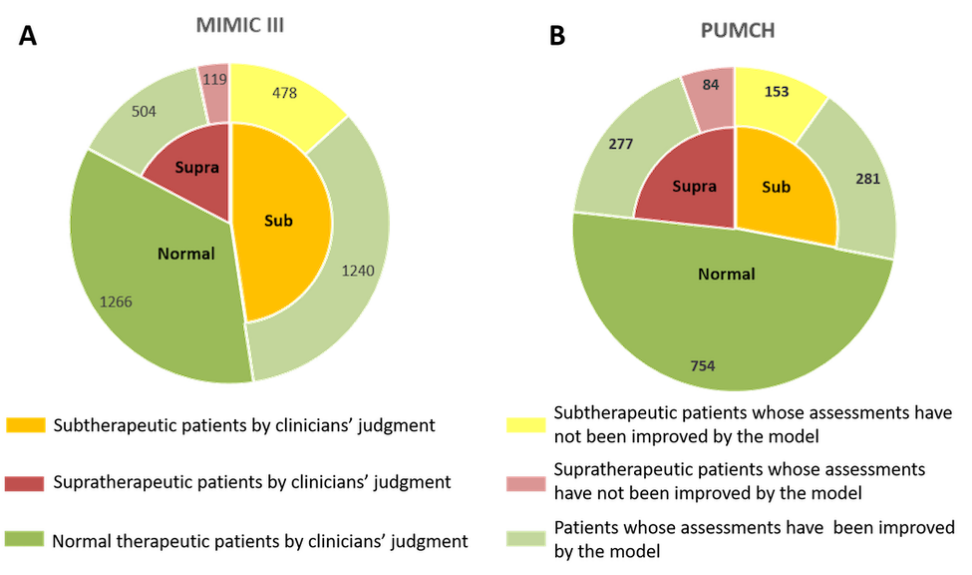
Recommend heparin dosage results in the MIMIC III (A) and PUMCH (B) data sets. MIMIC III: Multiparameter Intelligent Monitoring in Intensive Care III; PUMCH: Peking Union Medical College Hospital.

We compared our recommended dosage with the actual dosage for subtherapeutic samples (MIMIC III: n=1718; PUMCH: n=434) and supratherapeutic samples (MIMIC III: n=623; PUMCH: n=361). For 72.2 % (1240/1718) of the subtherapeutic samples in MIMIC III and 64.7% (281/434) of the subtherapeutic samples in PUMCH, our model recommended a higher heparin dosage than the clinicians did. Moreover, for 80.9% (504/623) of the supratherapeutic samples in MIMIC III and 76.7% (277/361) of the supratherapeutic samples in PUMCH, our model recommended a lower heparin dosage than the clinicians did. In Figure 4, the solid yellow, green, and red in the inner circles represent the subtherapeutic, normal, and supratherapeutic samples, respectively. The same shaded colors in the outer ring indicate that the recommended therapeutic state matches the inner circle, whereas fine green bars in the outer ring indicate the recommendation to increase the heparin dosage in subtherapeutic samples and to decrease the heparin dosage in supratherapeutic samples. The model recommendations may improve heparin treatment outcomes and may effectively reduce the time to optimal dosage in the clinical setting.

## Discussion

### Principal Results

In this study, we used a validated shallow neural network model for the clinical scenario of UFH infusion, which is commonly applied in the treatment and prevention of venous thromboembolism. To our knowledge, our study is the first practical validation of a machine learning–based model in the area of medication dosing optimization. We demonstrated the feasibility and efficiency of the proposed model in MIMIC III and in a local critical care database in a Chinese tertiary teaching hospital. Based on the calculated probabilities of individual circumstances, we proposed a UFH dosage recommendation for each record, and the comparison results show that the predicted recommended dosage can satisfactorily match the clinical practice.

### Comparison With Prior Work

As described in a previous study by Ghassemi using the MIMIC II database [14], a large variation appears in the initial dosing and the corresponding aPTT response, which suggests an underlying discrepancy in both the interprovider practice and patient factors. In the clinical scenario, to avoid this deviation and subsequent fluctuations and treatment-related risks, in our local practice, instead of empirically setting an initial dosage, a step-by-step pattern was adopted. The applicable dosing level was determined according to a series of continuously monitored aPTT results. Therefore, in this study, we used the steady state aPTT value after 8 h of heparin treatment instead of a single value. The results show that the machine learning–based model can effectively predict the aPTT response after the initial dosing and in a step-by-step pattern, which can contribute to decreasing the duration of the therapeutic regime and avoiding treatment-related risks. In our previous work, we demonstrated that the shallow neural network algorithm performed best compared to algorithms such as extreme gradient boosting, adaptive boosting, and support vector machine [11]. Based on our local clinical database and modified treatment pattern, we validated our previously developed model and demonstrated the applicability of this machine learning–based algorithm for UFH treatment. Despite having trained the machine learning models in our previous study on 2 public data sets, which mainly come from the well-known MIMIC and electronic ICU databases, they may not be generalizable to other institutions and populations. Nevertheless, our experience has shown that in a specific clinical scenario, the models can be smoothly migrated to a new data set after retraining, which reflects the flexibility and simplicity of machine learning algorithms. It is worth noting that validation on different data sets demonstrates the generalizability of the model, but the model will not necessarily have the same set of coefficients for each data set.

The interpretability problem remains an issue in the application of machine learning algorithms to the clinical setting. Interpretability can contribute to a physician making decisions based on numerous clinical variables rather than simply providing a prediction or description [22]. In this study, we calculated the importance of each feature in the shallow neural network model. As shown in Figure 3, the total heparin dosage, weight, initial aPTT value, creatinine value, and age are the 5 most important features for both data sets, although their specific ordering differs between data sets. In addition, as shown in Table 4, the average total heparin dosage increased successively from the subtherapeutic patients to the normal therapeutic patients and then to the supratherapeutic patients. It is reasonable that older age, lesser weight, and higher creatinine level tend to lead to supratherapeutic dosing. Conversely, younger age, higher weight, and lower creatinine level tend to lead to subtherapeutic dosing. This is consistent with clinical experience and can provide some practical support for clinicians. The feature importance analysis not only describes how the model works to a certain extent but can also prompt clinicians to pay more attention to important clinical variables. Although artificial neural network models are often regarded as black box models, we believe that the analysis and interpretation of these models will help understanding the model.

For each subtherapeutic or supratherapeutic patient, we recommended a total heparin dosage through the model. It is unreasonable to evaluate the recommended dosage with the model itself; therefore, we evaluated the rationality of the recommended dosage by comparing with the actual dosage given by the clinicians. It is noteworthy that in the MIMIC III data set, since the total heparin dosage is the most important feature, it contributed most to reasonable heparin dosage recommendation. In contrast, for the PUMCH data set, since the initial aPTT value is the most important feature and the total heparin dosage is relatively less important, the evaluation results of reasonable recommended heparin dosage are slightly low, as shown in Figure 4. Furthermore, the recommendation dosage results were initially evaluated in this study. For example, recommending increased dosage to subtherapeutic patients is evaluated as improved outcome; however, the recommended dosage may not definitely lead to a normal heparin treatment outcome—it may also lead to a supratherapeutic condition. We will further improve the evaluation method in our future studies.

### Limitations

This study has several limitations. First, our study was limited by its retrospective nature and the sources of the data. The performance of this machine learning–based model should be validated in clinical practice in order to provide valuable suggestions for treatment. Therefore, in addition to model optimization and cross-validation using more clinical databases, a well-designed, prospective, crossover clinical study should be performed. Second, our results were limited by the size of the populations in the clinical databases; a larger cohort might contribute to more accurate predictions and more precise recommendations for the steady dosing level. Considering the similarity of the data and logical structure, other clinical scenarios such as therapeutic drug monitoring, homeostasis balancing, and blood transfusion control could be appropriate applications of this model. Third, as described above, considering the different time span, treatment pattern, and patient characteristics of the MIMIC III and PUMCH clinical databases, such as 2 feature sets are not completely consistent, the optimal dosing level and the corresponding aPTT results differ in the 2 data sets. Nevertheless, these disparities did not cause the dose-effect relationship to differ in either the clinical practice or the machine learning algorithm, as demonstrated by the model performance and feature importance results. Although input features need to be adjusted or preprocessed when applied to the other data set, we still regarded that the prediction model and recommendation method provide a machine learning solution when applied to heparin outcome prediction and decision-making clinical scenarios. Furthermore, from the perspective of model development, our fully connected shallow neural network model is currently a static model that does not make use of dynamic time series data. In addition, we have not incorporated other available relevant features into the model, such as medical history, comorbidities, surgery history, and intake of medications other than heparin. In the future, with the goal of achieving better predictions of the outcome of heparin treatment and recommending more reasonable heparin dosages, we will build a dynamic model such as a recurrent neural network or a long short-term memory model and incorporate more features.

### Conclusions

Based on the machine learning model trained and validated in our previous work, this study aimed to further validate the model and its shallow neural network in a local clinical setting. We found that the data-driven machine learning method could be used effectively in the clinical scenario of UFH treatment with a step-by-step dosage pattern. The results provide support for predicting UFH treatment outcomes and recommending optimal UFH dosing to clinicians. We also evaluated the importance of each model feature to aid in the interpretation and understanding of the machine learning model.

## Appendix

Multimedia Appendix 1 Normal ranges and number of outliers for each feature of clinical interest.

Multimedia Appendix 2 Missing data imputation results.

Multimedia Appendix 3 Density functions of different features.

Multimedia Appendix 4 Proportions of each therapeutic level after dividing the data sets.

## Abbreviations

ALT: alanine aminotransferase
aPTT: activated partial thromboplastin time
AST: aspartate aminotransferase
ICU: intensive care unit
MIMIC III: Multiparameter Intelligent Monitoring in Intensive Care III
PUMCH: Peking Union Medical College Hospital
SOFA: sequential organ failure assessment
UFH: unfractionated heparin
