# Supplementary material for: A Clinical Prediction Model to Predict Heparin Treatment Outcomes and Provide Dosage Recommendations: Development and Validation Study
Source: J Med Internet Res. 2021 May 20;23(5):e27118. doi: 10.2196/27118 (PMC8176336; doi:10.2196/27118)
Supplement: Multimedia Appendix 1 [file jmir_v23i5e27118_app1.docx]

**Appendix I. Normal ranges and outliers**

We use [max(0,μ-3σ), μ+3σ] as the normal range and view data outside of this range as outliers. The results are shown in Table A1.

**Table A1.** Normal ranges and outliers in the two databases.

|  | Feature | Normal range | Outliers |
| --- | --- | --- | --- |
| MIMIC III | Weight | [0,171.8] | 42 |
|  | Initial aPTT value | [0,146.5] | 200 |
|  | Creatinine value | [0,6.5] | 103 |
|  | AST/ALT ratio | [0,8.4] | 29 |
|  | Total heparin amount | [0,45668.6] | 155 |
| PUMCH | Weight | [28.1,107.9] | 13 |
|  | Initial aPTT value | [0,78.0] | 25 |
|  | Creatinine value | [0,517.5] | 39 |
|  | ALT value | [0,1078.4] | 20 |
|  | Total heparin amount | [0,25.1] | 25 |
