# Supplementary material for: A Clinical Prediction Model to Predict Heparin Treatment Outcomes and Provide Dosage Recommendations: Development and Validation Study
Source: J Med Internet Res. 2021 May 20;23(5):e27118. doi: 10.2196/27118 (PMC8176336; doi:10.2196/27118)
Supplement: Multimedia Appendix 2 [file jmir_v23i5e27118_app2.docx]

**Appendix II. Missing data imputation results**

In both datasets, some SOFA scores were missing and the number of records are listed in Table A2. The missing values were interpolated using the 3-nearest neighbors algorithm.

**Table A2.** Number of missing values for each SOFA score.

|  | MIMIC III | PUMCH |
| --- | --- | --- |
| Coagulation SOFA score | 4 | 2 |
| Liver SOFA score | 1,351 | 7 |
| Cardiovascular SOFA score | 3 | 6 |
| Renal SOFA score | 0 | 7 |
