# Supplementary material for: A Clinical Prediction Model to Predict Heparin Treatment Outcomes and Provide Dosage Recommendations: Development and Validation Study
Source: J Med Internet Res. 2021 May 20;23(5):e27118. doi: 10.2196/27118 (PMC8176336; doi:10.2196/27118)

**Appendix III. Density functions of different features**

The density plots of the numerical features are shown in Figure A1.

**Figure A1.** Density plot of some numerical features


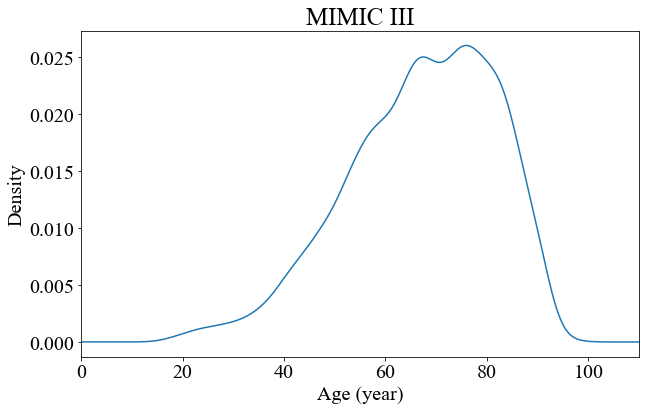

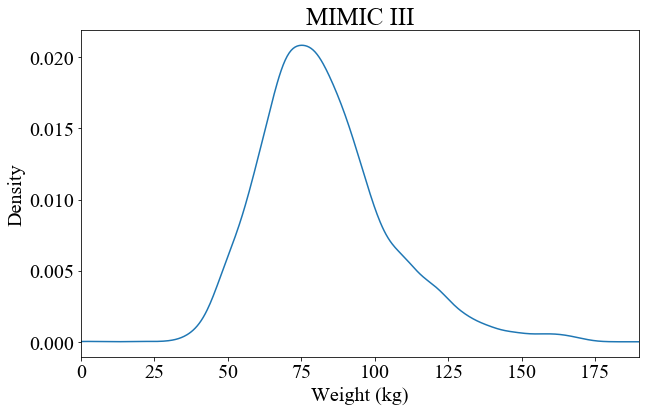


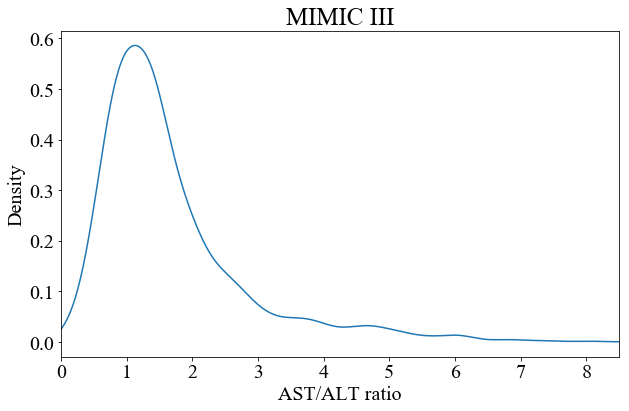

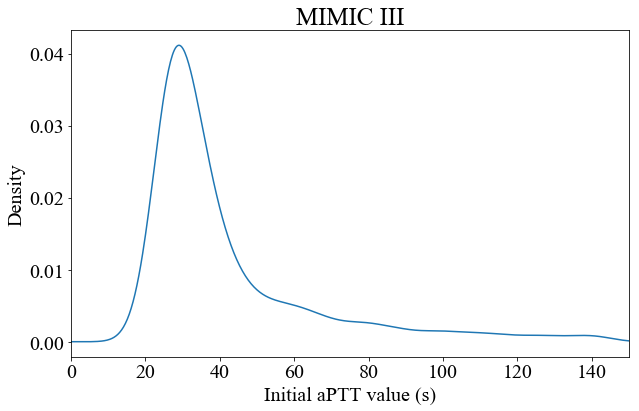


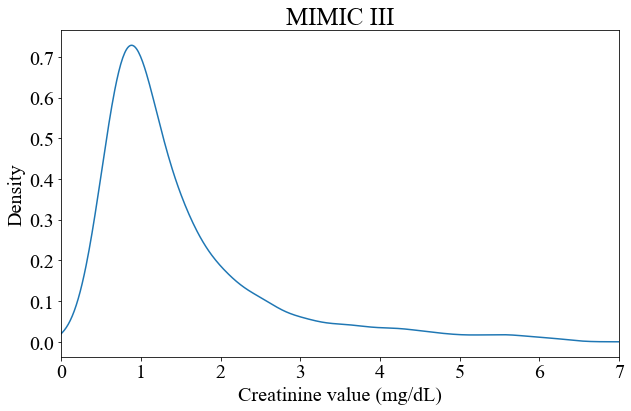

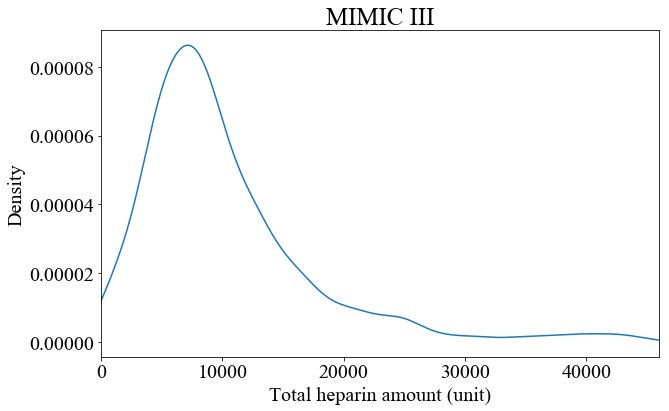


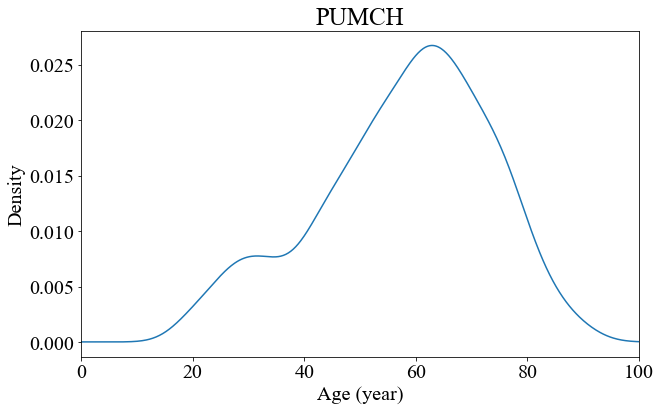

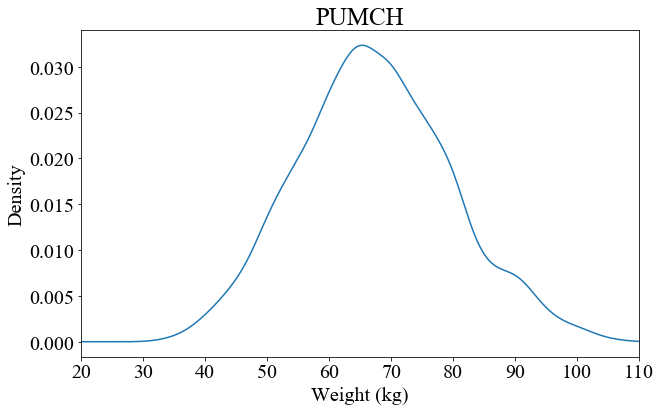


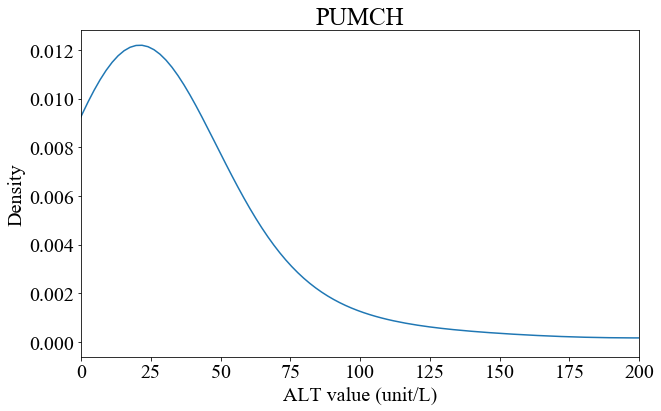

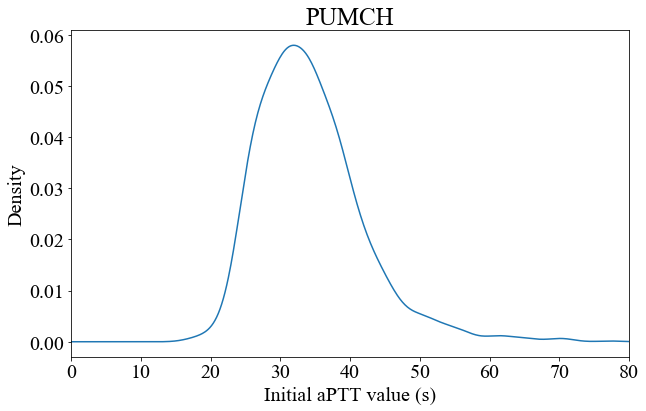

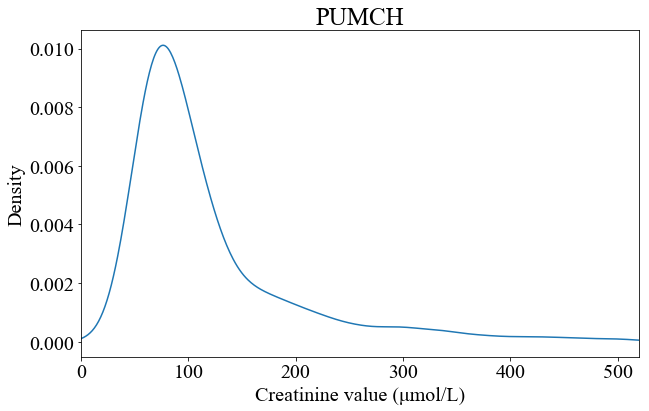

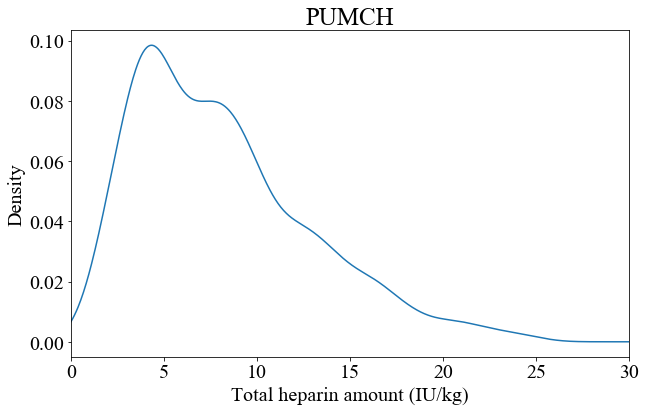

Supplement: Multimedia Appendix 3 [file jmir_v23i5e27118_app3.docx]
