# Supplementary material for: A Clinical Prediction Model to Predict Heparin Treatment Outcomes and Provide Dosage Recommendations: Development and Validation Study
Source: J Med Internet Res. 2021 May 20;23(5):e27118. doi: 10.2196/27118 (PMC8176336; doi:10.2196/27118)
Supplement: Multimedia Appendix 4 [file jmir_v23i5e27118_app4.docx]

**Appendix IV. Dataset Splitting**

We divided each dataset into a training set and a test set with an 80%/20% split. The proportion of subtherapeutic records, normal therapeutic records, and supratherapeutic records was maintained after the split, as shown in Table A3.

**Table A3.** Proportions of therapeutic levels after dividing the records into training and test sets.

|  | MIMIC III | | | PUMCH | | |
| --- | --- | --- | --- | --- | --- | --- |
|  | Training set | Test set | Total | Training set | Test set | Total |
| Sub | 1,374 | 344 | 1,718 | 347 | 87 | 434 |
| Nor | 1,012 | 254 | 1,266 | 603 | 151 | 754 |
| Sup | 498 | 125 | 623 | 288 | 73 | 361 |
| Total | 2,884 | 723 | 3,607 | 1,238 | 311 | 1,549 |
